# Densities, Excess Molar Volumes, and Thermal Expansion Coefficients of Aqueous Aminoethylethanolamine Solutions at Temperatures from 283.15 to 343.15 K

**DOI:** 10.1007/s10953-014-0175-2

**Published:** 2014-05-14

**Authors:** Marcin Stec, Adam Tatarczuk, Dariusz Śpiewak, Andrzej Wilk

**Affiliations:** Institute for Chemical Processing of Coal, Zamkowa 1, 41-803 Zabrze, Poland

**Keywords:** AEEA, Aminoethylethanolamine, Density, Excess properties, Thermal expansion coefficient

## Abstract

The densities of aqueous mixtures of aminoethylethanolamine (CAS #000111-41-1) were measured over the entire compositional range at temperatures of 283.15–343.15 K. The results of these measurements were used to calculate excess molar volumes and isobaric thermal expansion coefficients, and partial molar and apparent molar volumes and excess isobaric thermal expansion coefficients were subsequently derived. The excess molar volumes were correlated as a function of the mole fraction using the Redlich–Kister equation. Temperature dependences of the Redlich–Kister coefficients are also presented. The partial molar volumes at infinite dilution of AEEA in water were determined using two different methods. In addition, the solution density was correlated using a Joubian–Acree model. Aqueous solutions of AEEA exhibit similar properties to the aqueous solutions of other alkanolamines (like monoethanolamine) used in acid gas sweetening.

## Introduction

Aminoethylethanolamine (2-[(2-aminoethyl)amino]-ethanol), C_4_H_12_N_2_O) is used to make a derivative (hydroxyethyl ethylene urea) used as a wet-adhesion additive for latex paints, as a fabric softener added to textile materials, and as a dispersant detergent additive for fuel. Among other alkanoloamines, AEEA has recently become of interest as an alternative solvent for acid gas treatment. Aqueous alkanoloamines have been widely used for removal of acid gas impurities like sulfur dioxide or carbon dioxide in the petrochemical, chemical, and natural gas industries and for post-combustion carbon capture from flue-gases. Recent research has particularly focused on the latter topic.

The use of aqueous alkanolamines is probably the most promising technology for carbon capture in coal-fired power plants. However, using monoethanolamine (MEA), diethanolamine (DEA) or methyldiethanolamine (MDEA), which are common in the petrochemical and natural gas industries, would reduce power plant output by 25–35 % due to the high energy requirements of the technology [1]. Multiple research activities have focused on lowering energy requirements and searching for activators of the most popular amine, MEA [2]. Sterically hindered amines like AMP (2-amino-2-methyl-1-propanol), piperazine and its derivatives, different amine blends, and finally AEEA are constantly analyzed because of the potential for profit.

AEEA has been found to have absorption capacity, CO_2_ reactivity, and energy efficiency higher than of those of the industry standard MEA [3, 4], while its kinetics of CO_2_ removal are also promising [5]. Additionally, AEEA has a low vapor pressure, which would limit losses of the gas during post-combustion carbon capture [6].

Literature related to the physical properties of AEEA and its aqueous solutions is scarce: Mundhwa et al. [7] reported densities, viscosities and refractive indices of aqueous solutions of AEEA, while Ikada et al. [8] presented densities and refractive indices of the pure amine. Bindwal et al. [5] published density, viscosity and N_2_O solubility data for four AEEA solution concentrations (1.5–3.0 kmol·m^−3^). Similar N_2_O solubility data for pure AEEA and 30 wt-% AEEA solution have been presented by Ma’mun [3].

This study extends the small database of the physical properties of AEEA aqueous solutions by presenting densities and derived volumetric properties over the entire composition range. Particular attention was given to water and amine rich regions, because the volumetric properties of mixtures at infinite dilution are the most interesting from a thermodynamic point of view. Various volumetric properties like excess molar volume, apparent molar volume, partial molar volume, and excess thermal expansion coefficients were calculated and correlated with the Redlich–Kister equation. The calculated properties were compared with those of other alkanolamines and organic polar compounds. The conclusions drawn are consistent with literature sources.

In addition, the density of the solutions was correlated using the Jouyban–Acree model [9], which is especially useful during practical, engineering design. This simple model allows calculation of the density of binary aqueous AEEA mixtures at different temperatures and in any composition. The densities of pure AEEA and water, and only other three parameters, are required to estimate density, with average error of around 0.1 %.

## Experimental

Aminoethylethanolamine [CAS #000111-41-1, 2-[(2-aminoethyl)amino]-ethanol), 99 %] was purchased from Sigma–Aldrich and was used without further purification. The density of the pure substance was measured and compared to published values (Table 1). There is very good agreement between experimental and literature values. Deionized and double distilled water was used to prepare solutions.

**Table 1:** Density of AEEA at different temperatures at atmospheric pressure

Component	*T*/K	*ρ*/g·cm^−3^	
		This work	Literature
AEEA	283.15	1.03652	–
	293.15	–	1.02907^a^
	298.15	1.02529	1.02528^b^, 1.025^c^
	303.15	–	1.02153^b^, 1.02181^a^
	313.15	1.01398	1.01402^b^, 1.01407^a^
	328.15	1.00262	–
	343.15	0.99115	0.99115^b^

^a^Reference [8]

^b^Reference [7]

^c^Reference [3]

The solutions were prepared by weighing using a A&D HR-200 analytical balance with an accuracy of ± 10^−6 ^kg. Care was taken to minimize exposure to ambient air during sample preparation to avoid carbon dioxide absorption. Density measurements were conducted at least 24 h after sample preparations to ensure proper degassing and mixture equilibration. The densities of the solutions were measured at atmospheric pressure with an Anton Paar DMA5000 M density meter with an accuracy ± 5×10^−6^ g·cm^−3^ at a specific temperature, held constant with accuracy ±1×10^−3 ^K by the built-in thermostat. The density measurements were performed as follows: the cell of the apparatus was filled with approximately 3 cm^3^ of solution, then the measurement was started. After reaching equilibrium, the apparatus showed a constant value of density. The filling and measurement was repeated. If the difference between measurements was in agreement with the accuracy of the density meter, the final result was calculated, as the average of the measurements. The density meter was periodically calibrated during measurements on water and air at 303.15 K in accordance with manufacturer guidelines.

The uncertainty of the mole fraction does not exceeding 5.0 × 10^−5^. The uncertainty of excess molar volume and of thermal expansion coefficients values are less than 2.0 × 10^−5^ cm^3^·mol^−1^ and 5.0 × 10^−4^ kK^−1^, respectively.

## Results and Discussion

### Density and Excess Molar Volume of AEEA (2) + Water (1)

**Fig. 1:**
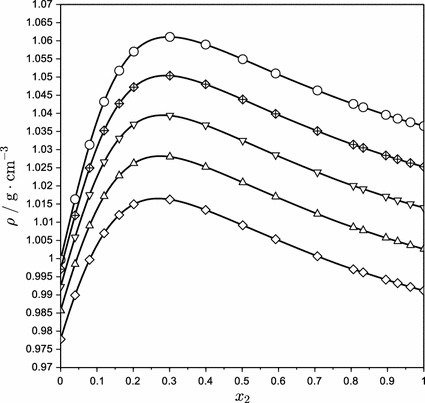
Densities of AEEA (**2**) + water (**1**) solution at various temperatures: *circle* 283.15 K;  298.15 K; *inverted triangle* 313.15 K; *triangle* 328.15 K; *diamond* 343.15 K. The* lines* serve only to join the data

The densities of aqueous mixtures of aminoethylethanolamine over the entire range of compositions at temperatures of 283.15–343.15 K are listed in Table 2 and plotted in Fig. 1. Starting from *x*_2_ = 0, the solution density increases sharply, reaches a maximum for AEEA concentrations around *x*_2_ = 0.3, and then smoothly decreases. Increasing temperature causes a density decrease and shifts the concentration of maximal density towards higher *x*_2_ values. Maximal density at 283.15 K occurs for approximately *x*_2_ = 0.25, while at 343.15 K for *x*_2_ = 0.3.

**Table 2:** Compositions, densities, excess molar volumes, and thermal expansion coefficients for AEEA (**2**) + water (**1**) mixtures at different temperatures

*x*_2_	283.15 K			298.15 K			313.15 K			328.15 K			343.15 K		
	*ρ* g·cm^−3^	*V* ^E^ cm^3^·mol^−1^	*α* kK^−1^	*ρ* g·cm^−3^	*V* ^E^ cm^3^·mol^−1^	*α* kK^−1^	*ρ* g·cm^−3^	*V* ^E^ cm^3^·mol^−1^	*α* kK^−1^	*ρ* g·cm^−3^	*V* ^E^ cm^3^·mol^−1^	*α* kK^−1^	*ρ* g·cm^−3^	*V* ^E^ cm^3^·mol^−1^	*α* kK^−1^
0.0000	0.99969	0.00000	0.086	0.99705	0.00000	0.258	0.99221	0.00000	0.384	0.98569	0.00000	0.485	0.97776	0.00000	0.57
0.0397	1.01635	−0.20443	0.206	1.01189	−0.20113	0.348	1.00585	−0.20339	0.454	0.99845	−0.20716	0.538	0.98988	−0.21128	0.61
0.0796	1.03136	−0.46922	0.319	1.02497	−0.45057	0.44	1.01753	−0.44422	0.529	1.00906	−0.44247	0.601	0.99967	−0.44284	0.662
0.1197	1.04322	−0.73921	0.414	1.03528	−0.70467	0.518	1.02660	−0.68646	0.595	1.01716	−0.67565	0.656	1.00697	−0.66795	0.709
0.1613	1.05177	−0.98349	0.491	1.04270	−0.93721	0.58	1.03308	−0.90859	0.647	1.02285	−0.88848	0.7	1.01201	−0.87236	0.746
0.2015	1.05703	−1.17341	0.546	1.04722	−1.11981	0.624	1.03698	−1.08488	0.682	1.02624	−1.05839	0.729	1.01496	−1.03573	0.769
0.3007	1.06107	−1.42803	0.624	1.05039	−1.37179	0.681	1.03940	−1.33163	0.724	1.02804	−1.29901	0.76	1.01627	−1.26936	0.79
0.3991	1.05897	−1.45639	0.659	1.04801	−1.40700	0.702	1.03675	−1.36908	0.736	1.02523	−1.33823	0.763	1.01339	−1.30954	0.787
0.5013	1.05493	−1.34976	0.68	1.04384	−1.30929	0.713	1.03250	−1.27695	0.74	1.02097	−1.25101	0.762	1.00918	−1.22741	0.781
0.5919	1.05101	−1.17909	0.695	1.03987	−1.14697	0.722	1.02853	−1.12167	0.743	1.01706	−1.10306	0.761	1.00534	−1.08403	0.777
0.7069	1.04632	−0.90088	0.709	1.03514	−0.87840	0.729	1.02378	−0.85884	0.745	1.01230	−0.84309	0.76	1.00065	−0.82839	0.773
0.8059	1.04261	−0.61707	0.717	1.03140	−0.60202	0.732	1.02006	−0.58934	0.745	1.00862	−0.57899	0.757	0.99704	−0.56925	0.769
0.8330	1.04169	−0.53667	0.719	1.03049	−0.52525	0.733	1.01917	−0.51565	0.745	1.00775	−0.50774	0.756	0.99619	−0.49951	0.767
0.8954	1.03960	−0.33909	0.722	1.02840	−0.33295	0.734	1.01710	−0.32860	0.745	1.00570	−0.32308	0.755	0.99420	−0.32023	0.765
0.9277	1.03856	−0.23197	0.724	1.02737	−0.22982	0.735	1.01605	−0.22503	0.745	1.00471	−0.22578	0.754	0.99322	−0.22361	0.764
0.9629	1.03755	−0.12072	0.727	1.02632	−0.11709	0.736	1.01502	−0.11638	0.745	1.00366	−0.11619	0.754	0.99218	−0.11467	0.763
1.0000	1.03652	0.00000	0.729	1.02529	0.00000	0.738	1.01398	0.00000	0.746	1.00262	0.00000	0.754	0.99115	0.00000	0.763

**Fig. 2:**
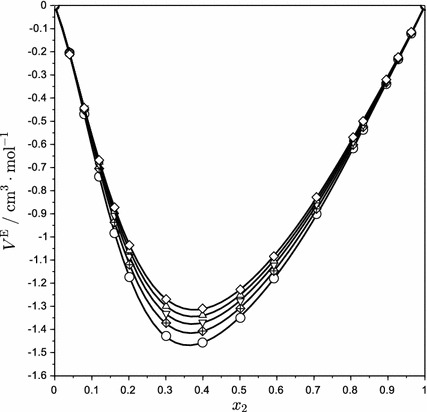
Excess molar volumes of AEEA (2) + water (1) solution at different temperatures: *circle* 283.15 K; , 298.15 K; *inverted triangle* 313.15 K; *triangle* 328.15 K; *diamond* 343.15 K; *solid lines*, Redlich–Kister equation

Excess molar volume is defined by the following equation:

1 $$ V^{\text{E}} = V_{\text{m}} - x_{1} V_{1}^{ * } - x_{2} V_{2}^{ * } $$

where *V*_m_ is the molar volume of the solution, *x*_1_ and *x*_2_ are the mole fractions of water and AEEA, respectively, and *V*_1_^*^ and *V*_2_^*^ are the molar volumes of pure water and AEEA. The molar volumes can be calculated from the density data and Eq. 1 takes the form:

2 $$ V^{\text{E}} = \frac{{x_{1} M_{1} + x_{2} M_{2} }}{\rho } - \frac{{x_{1} M_{1} }}{{\rho_{1}^{ * } }} - \frac{{x_{2} M_{2} }}{{\rho_{2}^{ * } }} $$

in which *M*_1_ and *M*_2_ are the molar masses of water and AEEA, *ρ* is the density of the mixture, and *ρ*_1_^*^ and *ρ*_2_^*^ represent the densities of pure water and AEEA, respectively. The excess molar volumes, calculated from Eq. 2, are listed in Table 2 and plotted in Fig. 2. The excess molar volumes of aqueous AEEA solutions show similar behavior to those of other alkanoloamines and are found to be negative for the whole concentration range. A small temperature effect is visible in Fig. 2: increasing the temperature makes the excess molar volume less negative. However, the minimum of the excess molar volume is almost temperature independent at concentration *x*_2_ = ~0.35. Similar results have been reported for other alkanolamines [10–17].

The excess molar volumes can be correlated using the Redlich–Kister equation:

3 $$ V^{\text{E}} = x_{2} \left( {1 - x_{2} } \right) \, \sum\limits_{n = 1} {C_{n} \left( {1 - 2x_{2} } \right)^{n - 1} } $$

The regressed values of coefficients *C*_*n*_ for each temperature are listed in Table 3, together with their residual standard deviations calculated from the following equation:

4 $$ \sigma = \left[ {\sum {\frac{{\left( {V_{ \exp }^{\text{E}} - V_{\text{calc}}^{\text{E}} } \right)^{2} }}{N - n}} } \right]^{{{1 \mathord{\left/ {\vphantom {1 2}} \right. \kern-0pt} 2}}} $$

where *N* is the number of data points and *n* is the order of fitting Redlich–Kister polynomial. Curves calculated with the Redlich–Kister equation are shown in Fig. 2 along with the experimental data.

**Table 3:** Redlich–Kister equation fitting coefficients and the residual standard deviation for AEEA + water mixtures

*T*/K	*C*_1_	*C*_2_	*C*_3_	*C*_4_	*C*_5_	*C*_6_	*σ*
283.15	−5.38456	−3.04642	−1.98758	−0.35485	3.63893	3.14454	0.00364
298.15	−5.22760	−2.88586	−1.67331	−0.03156	3.19468	2.59304	0.00282
313.15	−5.10336	−2.77551	−1.46166	0.13923	2.78997	2.17645	0.00250
328.15	−5.00595	−2.65865	−1.25834	0.10462	2.35996	1.99547	0.00214
343.15	−4.91262	−2.56262	−1.10657	0.10253	1.99280	1.76436	0.00227

The partial molar volume of each component is defined by:

5 $$ \bar{V}_{i} = \left( {\frac{\partial V}{{\partial n_{i} }}} \right)_{{T,p,n_{j} }} $$

Differentiation of Eq. 5 and combination with Eq. 1, as described by Wood and Battino [18], gives the following equations for the partial molar volume of water:

6 $$ \bar{V}_{1} = V^{\text{E}} + V_{1}^{ * } - x_{2} \left( {\frac{{\partial V^{\text{E}} }}{{\partial x_{2} }}} \right)_{p,T} $$

and for the partial molar volume of aminoethylethanolamine:

7 $$ \bar{V}_{2} = V^{\text{E}} + V_{2}^{ * } + \left( {1 - x_{2} } \right) \, \left( {\frac{{\partial V^{\text{E}} }}{{\partial x_{2} }}} \right)_{p,T} $$

The derivative of *V* ^E^ with respect to *x*_2_, present in Eqs. 6 and 7, can be calculated by differentiation of the Redlich–Kister equation (Eq. 3). After substitution of Eq. 3 into Eqs. 6 and 7 and the mentioned differentiation, one obtains the following equations for the partial molar volumes of water and AEEA, respectively:

8 $$ \bar{V}_{1} = V_{1}^{ * } + x_{2}^{2} \, \sum\limits_{n = 1} {C_{n} \left( {1 - 2x_{2} } \right)^{n - 1} } + 2x_{2}^{2} \, \left( {1 - x_{2} } \right) \, \sum\limits_{n = 1} {C_{n} \left( {n - 1} \right) \, \left( {1 - 2x_{2} } \right)^{n - 2} } $$

9 $$ \bar{V}_{2} = V_{2}^{ * } + \left( {1 - x_{2} } \right)^{2} \, \sum\limits_{n = 1} {C_{n} \left( {1 - 2x_{2} } \right)^{n - 1} } - 2x_{2}^{2} \, \left( {1 - x_{2} } \right) \, \sum\limits_{n = 1} {C_{n} \left( {n - 1} \right) \, \left( {1 - 2x_{2} } \right)^{n - 2} } $$

**Fig. 3:**
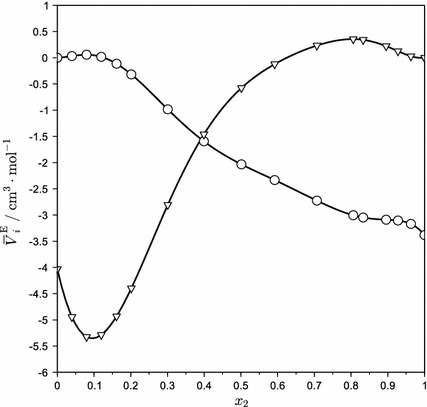
Excess partial molar volumes of water (*circle*) in AEEA and AEEA (*inverted triangle*) in water at 298.15 K calculated from Eqs. 8 and 9, respectively

To increase the features of the partial molar data, excess partial molar volumes were plotted instead of partial molar volumes. Curves for the partial molar excess volume $V_{1}^{\text{E}}$ of water in the AEEA and for the partial molar excess volume $V_{2}^{\text{E}}$ of AEEA in water at 298.15 K are shown in Fig. 3. The excess partial molar volumes were calculated by subtracting the pure component molar volumes *V*_1_^*^ and *V*_2_^*^ from the right hand sides of Eqs. 8 and 9, respectively. The curve for the partial molar volume of AEEA in water $V_{2}^{\text{E}}$ is similar to those of other polar organic compounds and shows a characteristic minimum [19]. The occurrence of such a minimum has been attributed to a balance between the effects of interstitial solution of amine molecules with accompanying enhancement of a clathrate-like structure in water, and its breakage with increasing amine concentration [20].

Another important property describing solution properties are the partial molar volumes of solution components at infinite dilution. Estimating them from the partial molar volumes is straightforward: setting *x*_2_ = 1 in Eq. 8 allows calculation of the partial molar volume of water at infinite dilution in AEEA:

10 $$ \bar{V}_{1}^{\infty } = V_{1}^{ * } + \sum\limits_{n = 1} {C_{n} \left( { - 1} \right)^{n - 1} } $$

Equally, setting *x*_2_ = 0 in Eq. 9 gives an equation for the partial molar volume of AEEA at infinite dilution in water:

11 $$ \bar{V}_{2}^{\infty } = V_{2}^{ * } + \sum\limits_{n = 1} {C_{n} } $$

The values of partial molar volume at infinite dilution are listed in Table 4 alongside the molar volumes of the pure components. The calculated values of the partial molar volume of AEEA at infinite dilution in water are in excellent agreement with Ref. [7]. The differences between the partial molar volumes of water at infinite dilution in AEEA are slightly higher, but, according to the authors, the corresponding reference data may be less reliable than those reported for AEEA [7].

**Table 4:** Partial molar volume and apparent molar volume of water (**1**) at infinite dilution in AEEA (**2**) and of AEEA (**2**) at infinite dilution in water (**1**), and molar volumes of pure species at different temperatures

*T*/K	$V_{{_{\varphi ,1} }}^{\infty }$	$\bar{V}_{1}^{\infty }$		*V*_1_^*^	$V_{{_{\varphi ,2} }}^{\infty }$	$\bar{V}_{2}^{\infty }$		*V*_2_^*^
		This work	Literature			This article	Literature	
283.15	14.8	14.5		18.0	96.8	96.5		100.5
298.15	14.9	14.7	15.0^a^	18.1	97.9	97.6	97.6^a^	101.6
313.15	15.0	14.8	15.0^a^	18.2	98.5	98.5	98.5^a^	102.7
328.15	15.1	14.9		18.3	99.2	99.4		103.9
343.15	15.3	15.1	15.5^a^	18.4	100.4	100.4	100.3^a^	105.1

Units are cm^3^·mol^−1^

^a^Reference [7]

Maham et al. [10] presented another approach to evaluation of the partial molar volume at infinite dilution. Instead of using the differentiation of the Redlich–Kister equation, they proposed graphical extrapolation of the apparent molar volume curves. The apparent molar volumes are defined as:

12 $$ V_{\varphi ,1} = V_{1}^{ * } + \frac{{V^{\text{E}} }}{{x_{1} }} = V_{1}^{ * } + \frac{{V^{\text{E}} }}{{1 - x_{2} }} $$

13 $$ V_{\varphi ,2} = V_{2}^{ * } + \frac{{V^{\text{E}} }}{{x_{2} }} $$

in which *V*_*φ*,1_ and *V*_*φ*,2_ are the apparent molar volumes of water in AEEA and of AEEA in water, respectively. Graphical extrapolation of the values of *V*_*φ*,1_ to *x*_1_ = 0 and those of *V*_*φ*,2_ to *x*_2_ = 0 gives the desired partial molar volumes at infinite dilution. Although graphical extrapolation and Redlich–Kister equation differentiation gave an evaluation of the same parameter, different symbols have been used to distinguish the method of calculation. The symbol $\bar{V}_{1}^{\infty }$ is used for partial molar volume of water at infinite dilution in AEEA calculated from Redlich–Kister coefficients, while $V_{{_{\varphi ,1} }}^{\infty }$ is that from graphical extrapolation. The worst disagreement between the values calculated in these two ways is 0.3 cm^3^·mol^−1^. The calculated results for both methods are summarized in Table 4.

The partial molar volumes of AEEA at infinite dilution $\bar{V}_{2}^{\infty }$ are smaller than the molar volumes of the pure amine *V*_2_^*^, and increase linearly with temperature. This is consistent with observations made for other alkanolamines (including MEA, DEA, TEA, and MDEA) by Hawrylak et al. [12]. As Maham et al. [10] suggest, volume contraction is caused by amine molecules occupying the voids that arise from the hydrogen bonded open structure of liquid water. Other confirmation of this conclusion arises from the theory of Hepler [21], who suggested that a positive sign of ${{\partial^{2} \bar{V}_{2}^{\infty } } \mathord{\left/ {\vphantom {{\partial^{2} \bar{V}_{2}^{\infty } } {\partial T^{2} }}} \right. \kern-0pt} {\partial T^{2} }}$ determines a “structure-making” while a negative sign determines a “structure-breaking” ability of the solute. A zero value of that derivative for AEEA at infinite dilution in water confirms that it has no effect on the ice-like structure of liquid water.

### Excess Thermal Expansion Coefficients

To extend understanding of the change in the structure of the solution during mixing, isobaric thermal expansions are calculated for every composition. The isobaric thermal expansion is defined by:

14 $$ \alpha = \frac{1}{{V_{\text{m}} }}\left( {\frac{{\partial V_{\text{m}} }}{\partial T}} \right)_{{p,x_{i} }} $$

Differentiation of *V*_m_ calculated from Eq. 1 with respect to *T* leads to:

15 $$ \left( {\frac{{\partial V_{\text{m}} }}{\partial T}} \right)_{{p,x_{i} }} = \left( {\frac{{\partial V^{\text{E}} }}{\partial T}} \right)_{{p,x_{i} }} + \frac{{\partial \left( {\sum\limits_{i = 1}^{2} {x_{i} V_{i}^{*} } } \right)}}{\partial T} $$

**Fig. 4:**
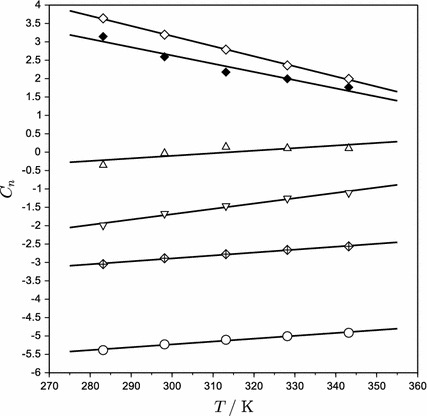
Temperature dependency of the Redlich–Kister coefficients *C*_*n*_: *circle* *C*_1_;  *C*_2_; *inverted triangle* *C*_3_; *triangle* *C*_4_; *diamond* *C*_5_; *solid lines* Eq. 17

Combining Eqs. 14 and 15 provides the final form of the equation for the isobaric thermal expansion coefficient:

16 $$ \alpha = \frac{1}{{V_{\text{m}} }}\left[ {\left( {\frac{{\partial V^{\text{E}} }}{\partial T}} \right)_{{p,x_{i} }} + \sum\limits_{i = 1}^{2} {\alpha_{i} x_{i} V_{i}^{*} } } \right] $$

where *α*_*i*_ is the thermal expansion coefficient of the pure component in the mixture. The thermal expansion coefficient for water *α*_1_ was regressed from data taken from Kell [22], while that of pure AEEA, *α*_2_, was regressed from the experimental density data collected in Table 1. Beside the thermal expansion coefficients of the pure components, differentiation of excess molar volume *V* ^E^ with respect to temperature is required for calculation of solution thermal expansion coefficients. It is straightforward to express Redlich–Kister correlation for *V* ^E^ as a function of temperature using the temperature dependency of the Redlich–Kister coefficients *C*_*n*_. These are a linear function of temperature (Fig. 4), so the temperature dependency can be expressed using polynomials as follows:

17 $$ C_{n} = \sum\limits_{i = 1}^{2} {d_{i} T^{i - 1} } $$

The values of the regressed temperature dependency parameter *d*_*i*_ are presented in Table 5. Appropriate linear correlations are also plotted in Fig. 4.

**Table 5:** Temperature dependency of the Redlich–Kister coefficients for the excess molar volume of AEEA (**2**) in water (**1**)

	*d*_1_	*d*_2_
*C*_1_	−7.56005	0.00777
*C*_2_	−5.28019	0.00797
*C*_3_	−6.04233	0.01451
*C*_4_	−2.20203	0.00701
*C*_5_	11.41100	−0.02751
*C*_6_	9.34502	−0.02239

Having the temperature dependent form of the excess molar volume *V* ^E^ and the values of the thermal expansion coefficients of the pure components, one can calculate the isobaric thermal expansion coefficients of mixtures using Eq. 16. The isobaric thermal expansion coefficients for aqueous solutions of AEEA are listed in Table 2 along with the data for pure AEEA.

Solution thermal expansion coefficients are presented in terms of excess values to emphasize solute–solvent influence on thermal expansion:

18 $$ \alpha^{\text{E}} = \alpha - \sum\limits_{i = 1}^{2} {\alpha_{i} \varphi_{i} } $$

where *φ*_*i*_ is the volume fraction of *i*-th component:

19 $$ \varphi_{i} = \frac{{x_{i} V_{i}^{*} }}{{\sum\limits_{i = 1}^{2} {x_{i} V_{i}^{*} } }} $$

**Fig. 5:**
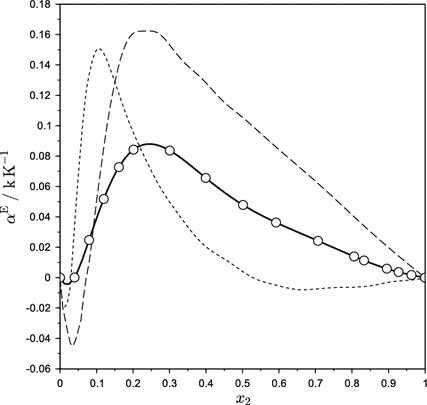
Excess thermal expansion coefficients of water (1) + AEEA (2) mixtures at 298.15 K from Eq. 18 compared with those of other polar solvents: *circle* AEEA solutions;* dashed line*, ethanol [20];* dotted line*, DMAPA [23]

Excess thermal expansion coefficients *α* ^E^ at 298.15 K are plotted in Fig. 5. The curve of *α* ^E^ is positive over the whole composition range except for a small negative loop in the water-rich region. There is a sharp increase in *α* ^E^ with increasing AEEA concentration, and the curve reaches its maximum near *x*_2_ = 0.25. At this concentration, the solution exhibits the highest deviation from its ideal mixing value by an order of around 10 %. Further increase in AEEA concentration causes gradual decrease of *α* ^E^. This characteristic is consistent with results reported for aqueous solutions of 3-dimethylamino propylamine (DMAPA)—another example of a diamine [23]. This behavior seems common also for other organic polar solvents like ethanol or 1-propanol, as presented by Benson et al. [20]. The excess thermal expansion coefficients *α* ^E^ show no distinct temperature dependency.

### Density Correlation with Jouyban–Acree Model

The Jouyban–Acree model can be used to correlate the density of binary mixtures as a function of mixture composition and temperature [9]. The density can be calculated using the expression:

20 $$ \ln \rho_{T} = x_{1} \ln \rho_{1,T}^{*} + x_{2} \ln \rho_{2,T}^{*} + J_{0} \left[ {\frac{{x_{1} x_{2} }}{T}} \right] + J_{1} \left[ {\frac{{x_{1} x_{2} \left( {x_{1} - x_{2} } \right)}}{T}} \right] + J_{2} \left[ {\frac{{x_{1} x_{2} \left( {x_{1} - x_{2} } \right)^{2} }}{T}} \right] $$

where *ρ*_*T*_, $\rho_{1,T}^{*},$and $\rho_{2,T}^{*}$ are the mixture density and the densities of pure water and the pure amine at temperature *T*, respectively, and *J*_*i*_ are the model constants regressed from the experimental density data. Despite the low number of parameters, this model works very well: the average error for the data listed in Table 2 does not exceed 0.1 %. The regressed parameters are listed in Table 6.

**Table 6:** Jouyban–Acree model fitting coefficients for the densities of AEEA(**2**) + water(**1**) mixtures at 283.15–343.14 K

Temperature range	*J*_o_	*J*_1_	*J*_2_	Average error
283.15–343.15 K	37.616	47.949	31.252	0.1 %

## Conclusions

Densities for aqueous mixtures of aminoethylethanolamine at temperatures of 283.15–343.15 K were measured for the entire compositional range. The excess molar volumes *V* ^E^ of the mixtures are negative at all of the temperatures. The partial molar volumes at infinite dilution $\bar{V}_{i}^{\infty }$ of both water in AEEA and AEEA in water are lower than the corresponding molar volumes of the pure species. The excess thermal expansion coefficients of water + AEEA mixtures are positive at all of the temperatures except for a small loop in the water rich region (*x*_2_ ~ 0.05) where *α* ^E^ is negative. The results show the ability of AEEA to occupy voids in the ice-like water structure. No effect of AEEA on the structure of water was found, based on Hepler’s theory. Our conclusions are consistent with results presented in the literature for other alkanolamines.
